# The Holin-Endolysin Lysis System of the OP2-Like Phage X2 Infecting *Xanthomonas oryzae* pv. *oryzae*

**DOI:** 10.3390/v13101949

**Published:** 2021-09-28

**Authors:** Zhifeng Wu, Yang Zhang, Xinyang Xu, Temoor Ahmed, Yong Yang, Belinda Loh, Sebastian Leptihn, Chenqi Yan, Jianping Chen, Bin Li

**Affiliations:** 1 State Key Laboratory of Rice Biology and Ministry of Agriculture Key Laboratory of Molecular Biology of Crop Pathogens and Insects, Institute of Biotechnology, Zhejiang University, Hangzhou 310058, China; 21916082@zju.edu.cn (Z.W.); 0618151@zju.edu.cn (Y.Z.); 12016074@zju.edu.cn (X.X.); temoorahmed@zju.edu.cn (T.A.); 2 State Key Laboratory for Managing Biotic and Chemical Threats to the Quality and Safety of Agro-Products, Institute of Virology and Biotechnology, Zhejiang Academy of Agricultural Sciences, Hangzhou 310021, China; yangyong@zaas.ac.cn (Y.Y.); yanchengqi@zaas.ac.cn (C.Y.); 3 University of Edinburgh Institute, Zhejiang University, Hangzhou 314400, China; belinda.loh@intl.zju.edu.cn (B.L.); leptihn@intl.zju.edu.cn (S.L.); 4 Institute of Plant Virology, Ningbo University, Ningbo 315211, China

**Keywords:** transmembrane domain, endolysin, holin, morphological change, lysis, phage

## Abstract

Most endolysins of dsDNA phages are exported by a holin-dependent mechanism, while in some cases endolysins are exported via a holin-independent mechanism. However, it is still unclear whether the same endolysins can be exported by both holin-dependent and holin-independent mechanisms. This study investigated the lysis system of OP2-like phage X2 infecting *Xanthomonas oryzae* pv. *oryzae*, causing devastating bacterial leaf blight disease in rice. Based on bioinformatics and protein biochemistry methods, we show that phage X2 employs the classic "holin-endolysin" lysis system. The endolysin acts on the cell envelope and exhibits antibacterial effects in vitro, while the holin facilitates the release of the protein into the periplasm. We also characterized the role of the transmembrane domain (TMD) in the translocation of the endolysin across the inner membrane. We found that the TMD facilitated the translocation of the endolysin via the Sec secretion system. The holin increases the efficiency of protein release, leading to faster and more efficient lysis. Interestingly, in *E. coli*, the expression of either holin or endolysin with TMDs resulted in the formation of long rod shaped cells. We conclude that the TMD of X2-Lys plays a dual role: One is the transmembrane transport while the other is the inhibition of cell division, resulting in larger cells and thus in a higher number of released viruses per cell.

## 1. Introduction

Phages (or bacteriophages) are viruses that specifically infect bacteria [1]. Most double-stranded DNA (dsDNA) tailed phages (i.e., Caudovirales) make use of the canonical holin-endolysin system to release progeny virions during the final stages of the lytic life cycle, resulting in lysis and thus killing the host. Most of the current research on the holin-endolysin system aimed to develop such proteins as therapeutics for biotechnological or for medical use, as endolysins have been shown to be effective antimicrobial agents [2,3,4,5]. Its use in medicine, food and agriculture is being extensively explored as an alternative to chemical treatments, which addresses concerns regarding safety, environmental burden and antimicrobial resistance of antibiotic substances. Thus, alternatives such as the use of bacteriophages or their proteins as bacterial biocontrol agents has attracted much attention in recent years [6,7,8,9,10]. For example, endolysins have been shown to be effective in eliminating or reducing the colonization of *Streptococcus pyogenes* in the respiratory tract of mice, thereby reducing the occurrence of related diseases [11]. As a potential biological control agent in various food processing environments, the endolysin of a *Staphylococcus aureus* phage has been shown to effectively remove *Staphylococcus* biofilms on the surface of food [12].

As part of the lysis system, endolysins are first produced and accumulated in the cytoplasm in the late stage of the lytic cycle of phages, where the proteins are unable to cross the cytoplasmic membrane to exert their function on the peptidoglycan layer [4]. Holins are small membrane proteins that facilitate lysis when they oligomerize [13], resulting in the formation of pores in the cytoplasmic membrane which in turn allows the release of endolysin into the periplasm [14,15]. Here, the endolysins which are peptidoglycan hydrolases start to digest the peptidoglycan layer of the bacterial cell wall. This process eventually leads to lysis of the cell due to the osmotic pressure difference between the cell and the surrounding environment. A holin-GFP fusion of the lambda phage protein was found evenly distributed in the inner membrane until the protein reached a critical concentration [16]. Once a certain threshold has been reached, large aggregates or “holin rafts” are formed, creating large holes in the inner membrane allowing the leakage of the cytosolic content (containing the endolysin) into the periplasm [16]. In some phages, anti-holins exist that were found to form heterodimers with holin and are thought to prevent the premature formation of holin rafts to suppress lysis of bacteria when phage assembly is not yet completed [16].

Holin-independent mechanisms are also found in the case of SAR-endolysin, which possesses a signal-arrest-release (SAR) domain that is necessary and sufficient for the bacterial export system to release endolysins into the periplasm without the help of holins [15,17,18]. The holin-independent endolysins such as those from phages P1, 21, and phiKMV with SAR domains are secreted to the periplasm via the Sec pathway where they induce cell lysis [19,20]. The SAR domain-containing endolysins are anchored to the cytoplasmic membrane in an inactive form until holins lead to the collapse of the membrane proton motive force. Interestingly, we previously showed that a transmembrane domain (TMD) facilitates endolysin transport via the Sec pathway in phage AP1 despite a putative “holin-endolysin” system being present in the phage genome [21]. These results indicate that both, the TMD and the holin, contribute to the transport of Lys, depending on the protein (and the phage). It remains unclear whether the TMD and holin can facilitate the transport of the same Lys. Addressing this question will help us to understand the evolution of the lysis system of the OP2-like phage family.

In this study, we identified a "holin-endolysin" lysis cassette by examining the genome of the OP2-like phage X2 and characterizing the phage release mechanism employed by the phage. X2 infects *Xanthomonas oryzae* pv. *oryzae* (Xoo), which causes devastating bacterial leaf blight disease in rice. We also examined the lysis activity and cell morphology changes during phage lysis when co-expressing either lysis protein or both together employing a variety of methods, including fluorescence and electron microscopy. Our study contributes to the understanding of the lysis mechanism used by phages, which may also allow us to potentially exploit these enzymes for infection prevention and biocontrol.

## 2. Materials and Methods

### 2.1. Bacteria, Plasmids and Growth Conditions

Xoo strain C2 and its phage X2 were isolated from diseased rice leaves as described in our previous studies [22,23], which were routinely cultured in nutrient agar medium and water, respectively. Plasmids used in this study are described in Table 1. The lysis related genes were expressed in *Escherichia coli* BL21 (DE3) (Vazyme Biotech), which were grown on Lysogeny Broth (LB) at 37 °C, 200 rpm, unless stated otherwise. Kanamycin (Km) was added to LB culture medium at 50 μg/mL to select for pET28a and its recombinant plasmids. Ampicillin (Amp) was added to the culture medium at 100 μg/mL to select for pETDuet-1 and its recombinant plasmids. To express genes under the control of the T7 promoter, IPTG (isopropyl-β-D-thiogalactopyranoside) stock solution with the initial concentration of 0.2 mol/L was added to obtain a final concentration of 1.0 mmol/L unless otherwise statement. Optical density of bacteria was determined using a spectrophotometer (Lambda35 UV/VIS; Perkin Elmer).

### 2.2. Genome Sequencing and Phylogenetic Analysis of Phage X2

Genomic DNA of phage X2 was extracted using a λ phage genome kit (Sangon Biotech, Shanghai, China). Sequencing of the X2 genome was conducted using the novogene platform (China). The X2 phage genome was annotated using the RAST (Rapid Annotation using Subsystem Technology) database (https://rast.nmpdr.org/rast.cgi; accessed on 5 November 2020). The identified open reading frames (ORFs) were further validated using BlastP of NCBI. Using the X2 phage genome and TERL (terminase large subunit), phylogenetic trees were constructed using MEGA7 software.

### 2.3. In Silico Genome-Wide Analysis of Lysis System

ClustalW2 was used to align the amino acid sequence of phage X2-Lys with homologous proteins. TMD analysis and signal peptide prediction were performed by TMHMM-2.0 (https://services.healthtech.dtu.dk/service.php?TMHMM-2.0; accessed on 29 July 2020) and SignalP-5.0 Server (http://www.cbs.dtu.dk/services/SignalP/; accessed on 29 July 2020). The structure of X2-Lys was predicted using Phye2 (http://www.sbg.bio.ic.ac.uk/phyre2/html/page.cgi?id=index; accessed on 9 November 2020). The conserved domain was predicted using the NCBI Conserved Domain Database. Promoter was predicted by using Bprom (http://www.softberry.com/berry.phtml?topic=bprom; accessed on 25 November 2020).

### 2.4. Construction of Recombinant Plasmids

To construct plasmid pET28a-Lys, the Lys gene of X2 phage was PCR amplified using X2 genomic DNA and primers 28a-Lys-F and -R (Table 2). The X2-Lys fragment was then cloned into pET28a using restriction enzymes, BamHI and HindIII. The *E. coli* BL21 (DE3) was transformed with recombinant plasmids by heat shock method [24]. This method was used to construct pET28a-Hol, pET28a-Lys-TMD, pETDuet-Lys, pETDuet-Hol and pETDuet-Lys-Hol. Transmembrane domain (TMD) was cloned from a known endolysin of LysAP [21].

### 2.5. Growth Measurement

The influence of X2-Lys and X2-Hol on bacterial growth was determined by comparison of the bacterial numbers, which was performed by measuring the optical density at 600 nm (OD600) using a microplate photometer based on a previously described method with minor revisions [25]. In brief, bacterial suspension was prepared by inoculating the freshly grown overnight culture into 5 mL of LB broth to obtain an initial concentration (OD600 = 0.4), while induction was initiated by adding IPTG stock solution of 0.2 mol/L into bacterial suspension to obtain a final concentration of 1.0 mmol/L and incubating at 20 °C, 200 rpm. The OD600 value was measured after 0, 1.5, 3, 6, 12, and 24 h of incubation. LB broth without bacteria was used as the negative control. The experiment was repeated three times with three replicates for each treatment.

### 2.6. Live/Dead Cell Staining and Flow Cytometry Observation

Bacterial lysis caused by the expression of X2-Lys and X2-Hol was determined by live/dead cell staining and flow cytometry analysis. Briefly, induction was initiated by adding IPTG stock solution of 0.2 mol/L into bacterial suspension (OD600 = 0.4), which was prepared as described above. After 12 h and 24 h of induction at 20 °C, 200 rpm, bacterial pellets were harvested by centrifugation at 5000× *g* for 10 min followed by three washes with 0.1 M phosphate-buffered saline (PBS). Live/dead staining assay was conducted using the BacLight bacterial viability kit (Invitrogen) as described by Masum et al. [26]. The kit includes two nucleic acid stains, (i) a red-fluorescent (propidium iodide stain, PI) for dead bacteria, and a green fluorescent (SYTO 9 stain) for live bacteria. Fluorescence was detected using an inverted confocal microscope (Leica-SP8, Heidelberg, Germany). Flow cytometry assays were carried out as described by Wu et al. [27] with minor modifications; bacterial cells were stained with PI solution at a final concentration of 50 mg/L for 20 min in the dark, washed two or three times with 0.1 M PBS, and then observed under the FACSVerse cytometer (BD Biosciences, San Jose, CA, USA).

### 2.7. Detection of β-Galactosidase Activity and Efflux of the Nucleic Acid

As described above, induction was initiated by adding IPTG into bacterial suspension (OD600 = 0.4) and then incubated at 20 °C for 6 h, 12 h and 24 h, respectively. After centrifugation at 12,000 rpm for 5 min, a 500 μL aliquot of extracellular supernatant was added with 100 μL of ortho-Nitrophenyl-β-galactoside (ONPG) (20 mM). The mixture was incubated in a 45 °C water bath for 30 min and the reaction was stopped by adding 600 μL Na_2_CO_3_ (0.5 mM). The β-galactosidase activity was determined by measuring the OD value at 420 nm (OD420) using a microplate photometer. The efflux of nucleic acid was determined as described by Wu et al. [27] by using a spectrophotometer (Thermo-Fisher Scientific, Waltham, MA, USA) to measure the amount of nucleic acid present in the supernatant, which were prepared by removing the above-mentioned IPTG induced bacteria through a 0.22 µm filter.

### 2.8. Transmission Electron Microscopic (TEM) Observation

Bacterial sample preparation for TEM was conducted as described by Abdallah et al. [28] with some revision. Briefly, bacteria were collected by centrifugation at 5000× *g* for 5 min, then washed three times with 0.1 M PBS solution followed by fixing with 2.5% (*v*/*v*) glutaraldehyde. The samples were then stained with 1% (*w*/*v*) osmium tetroxide in 0.1 M PBS for 1 h at room temperature, then washed three times with 0.1 M PBS. Following this, the samples were dehydrated stepwise over a range of ethanol solutions (70%, 80%, 90%, 95% and 100% *v*/*v*) with each step lasting for 15 min at room temperature. Dehydrated samples were embedded in Epon 812, a low-viscosity embedding resin. The morphological changes to bacterial cells were observed using a TEM (JEM-1230, JEOL, Akishima, Japan) according to the operating methods.

### 2.9. Purification and Antibacterial Effect of X2-Lys

The recombinant strains were incubated in LB (with 50 µg/mL kanamycin) at 37 °C, 200 rpm and then its concentration was adjusted to OD600 = 0.6. After 16 h induction at 20 °C, 200 rpm with 1 mM IPTG and centrifugation, the bacterial pellets were collected, resuspended in 0.1 M PBS and lysed by sonication (400 W, ultrasound 4 s, interval 8 s). Following the centrifugation of bacteria at 8000× *g* for 30 min at 4 °C, the target protein in lysate supernatant was purified using ProteinIso™ Ni-NTA Resin (Transgen Biotech, China) according to the instructions. After concentration using a 10.0 kDa cut-off ultrafiltration tube, the purified protein was subjected to both SDS-PAGE analysis and Western blotting detection.

The antibacterial effect of recombinant protein X2-Lys on Xoo strain C2 was determined as previously described [11,29,30]. Briefly, in a 96-well plate each well was filled with 100 μL of bacterial suspension (OD600 = 1.15) and 100 μL endolysin X2-Lys (0.32 mg/mL). X2-Lys was produced as described above, while bacterial suspensions were prepared by incubating cell pellets from overnight culture (0.6 of OD600) with 100 mM EDTA for 5 min at room temperature, then centrifuged at 8000 rpm, 4 °C for 10 min, washed three times with 0.1 M PBS, and then resuspended in 50 mM Tris/HCl, (pH 8.2, containing 0.1% Triton X-100). OD450 was measured using a microplate photometer. Lysozyme (0.32 mg/mL) and PBS buffer (0.1 M) were used as positive and negative control, respectively. The experiment was repeated three times with three replicates for each treatment.

### 2.10. Extraction and Detection of Membrane Proteins

Bacteria for the extraction of membrane proteins were prepared by inoculating 5 mL of LB with freshly grown overnight culture of Xoo strain C2 to OD600 = 0.4, and then centrifuged at 12,000 rpm, 4 °C for 5 min. Membrane proteins were extracted from the harvested cell pellets using a Bacterial Membrane Protein Extraction Kit (Bestbio, Shanghai, China) according to the manufacturer’s instructions. The extracted protein samples were then analyzed by both SDS-PAGE and Western blotting.

### 2.11. Statistical Analysis

The ANOVA test was analyzed by Graphpad prism (version 8.0). Levels of significance (*p* < 0.05) of main treatments and their interactions were calculated by analysis of variance after testing for normality and variance homogeneity.

## 3. Results and Discussion

### 3.1. Analysis of Phage X2 Genome

**Figure 1:**
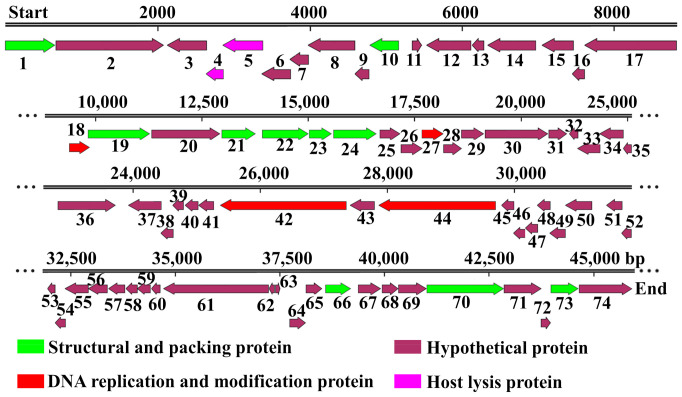
Genome map of phage X2. Double black lines indicate the genome. Numbers above indicate nucleotide position. Block arrows indicate open reading frames (ORFs) and the direction of each read. Each ORF is numbered in running order as indicated by numbers below.

**Figure 2:**
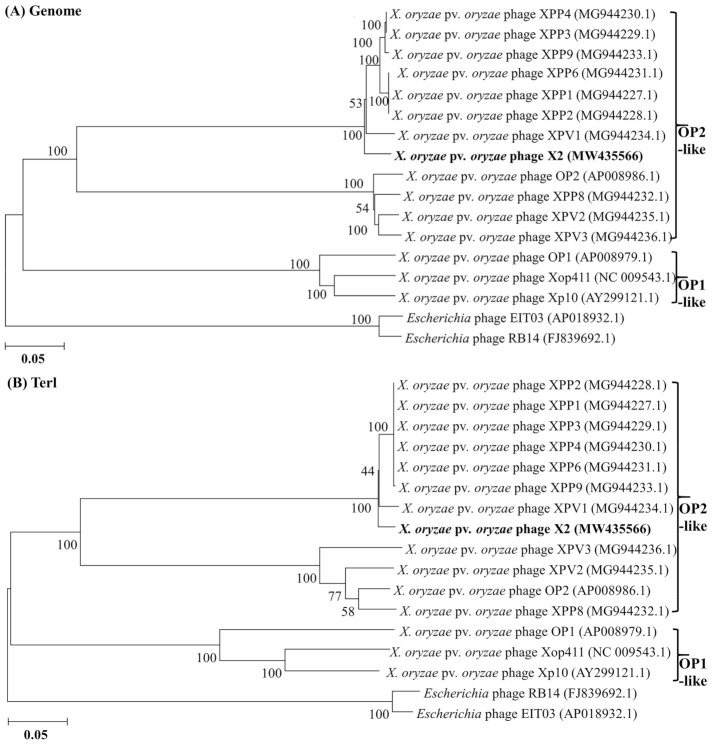
Neighbor-joining phylogenetic tree analysis of X2 phage. (**A**) Genome. (**B**) TERL. Bootstrap values of 1000 replications. *Escherichia* phage was used as the out-group.

After DNA isolation and sequencing, we analyzed the bacteriophage X2 genome which contains 45,966 nucleotides and has a 61.27% GC content. Based on the NCBI BlastP and RAST annotations, 74 ORFs were predicted, of which 16 ORFs can be assigned to proteins with known functions (Table S1). The ORFs can be divided into four main categories: hypothetical proteins, host lysis proteins, phage structure and packing proteins, phage DNA replication and modification proteins (Figure 1). Through a phylogenetic analysis of the X2 phage genomic sequence, X2 phages clustered with Xoo phage OP2 and other OP2-like phages, such as XPP1, XPP2, XPP3, XPP4, XPP6, XPP8, XPP9, XPV1, XPV2 and XPV3, and were well separated from the other Xoo phages OP1 and OP1-like phages, such as Xop411 and XP10 (Figure 2A). Similar results were obtained from a phylogenetic analysis of *Terl* gene sequence encoding the terminase large subunit (Figure 2B), which is considered to be a highly conserved gene [31,32].

A comparison of the available genomic data of Xoo phages revealed that phage X2 should be classified as an OP2-like phage due to the greater similarity phage X2 has with OP2-like phages rather than OP1-like phages (Table 3). In general, OP2-like phages have a larger genome size, ORF number and higher G+C content than that of OP1-like phages. For example, the G+C content of OP2-like phages is approximately 60–64%, while the G+C content OP1-like phages is approximately 51–52%. In addition, even though all the identified phages of Xoo belong to order of *Caudovirales*, there are differences in morphology between OP1- and OP2-like phages. Indeed, phage OP2 including X2 belongs to *Myoviridae* [33,34], while the OP1-like phages such as Xop411 and Xp10 belong to *Siphoviridae* [35,36].

### 3.2. In Silico Identification of Phage X2 Lysis System

**Figure 3:**
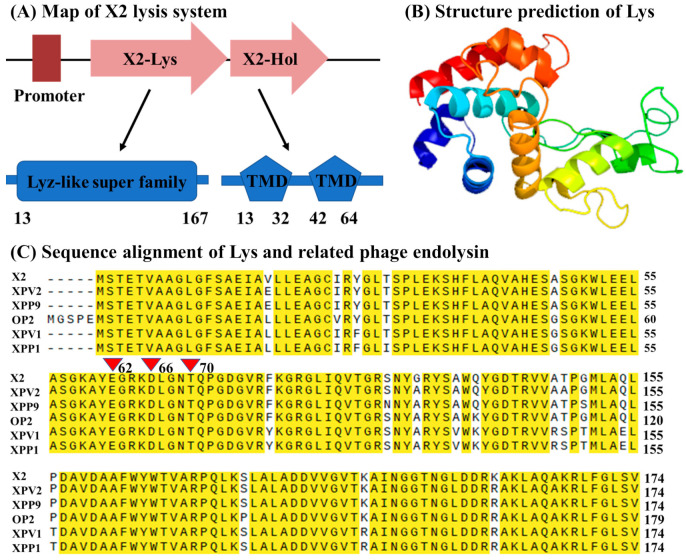
In silico analysis of phage X2 lysis system. (**A**) Schematic representation of the X2 lysis system. Numbers indicate the amino acid position. TMD: transmembrane domain. (**B**) Three-dimensional structure prediction of X2-Lys. The confidence is 100%, the coverage is 93%. (**C**) Sequence alignment of X2-Lys with that of phage XPV2, XPP9, OP2, XPV1 and XPP1 (Accession no. AVO24273.1, AVO24087.1, YP_453642.1, AVO24202.1 and AVO23647.1, respectively). Red triangles (62, 66 and 70) are the key residues (Glu11, Asp20 and Thr26) in the catalytic triad of phage T4 lysozyme, respectively.

The lysis system of X2 is predicted to consist of two proteins, endolysin (X2-Lys) and holin (X2-holin), respectively, with a putative TTGCAC-19nt-TGGTAAGCT promoter located upstream of the X2-Lys ORF; the Lyz-like super family domain was identified between amino acid 13 and 167 of X2-Lys (Figure 3A). Signal peptide- and transmembrane domain prediction indicate that X2-Lys does not have either, suggesting that holin may be required for this protein to translocate across the inner membrane. However, initially, we were unable to identify a gene coding for a holin by employing RAST and Blastp. It is well known that the holin gene is normally in the genomic vicinity of the endolysin gene and contain at least one transmembrane domain [37,38]. Thus, we searched for a transmembrane domain of a total of 20 ORFs in the vicinity of the putative endolysin. Only one protein (X2-Hol) with a length of 75 amino acids contains two transmembrane domains, with its N- and C-terminus predicted to be located in the cytoplasm (Figure 3A). Holins are divided into three categories: Type I holins have three transmembrane domains, while type III holins have one transmembrane domain. Type II holins have two transmembrane domains, and both N- and C-terminus are in cytoplasm [13,39]. Therefore, X2-Hol is likely to belong to the type II holins.

The Protein Fold Recognition Server Phyre2 predicted X2-Lys to have helical structures (Figure 3B), which are characteristic of the lysozyme family [3,5]. X2-Lys that contains 174 amino acids displayed the highest homology to that of the five other Xoo phages (Figure 3C). Interestingly, key residues (Glu11, Asp20 and Thr26) in the catalytic triad of T4 lysozyme [40] were found in X2-Lys (Glu62, Asp66 and Thr70) although there is no sequence similarity between X2-Lys and lysozyme from the T4 phage. To date, at least four types of endolysins including lysozymes, transglycosidases, amidases and endopeptidases have already been identified [41]. Therefore, we conclude that the X2-Lys is a lysozyme endolysin.

### 3.3. Phage X2 Lysis System Similar to OP2- Other Than OP1-Like Phages

**Figure 4:**
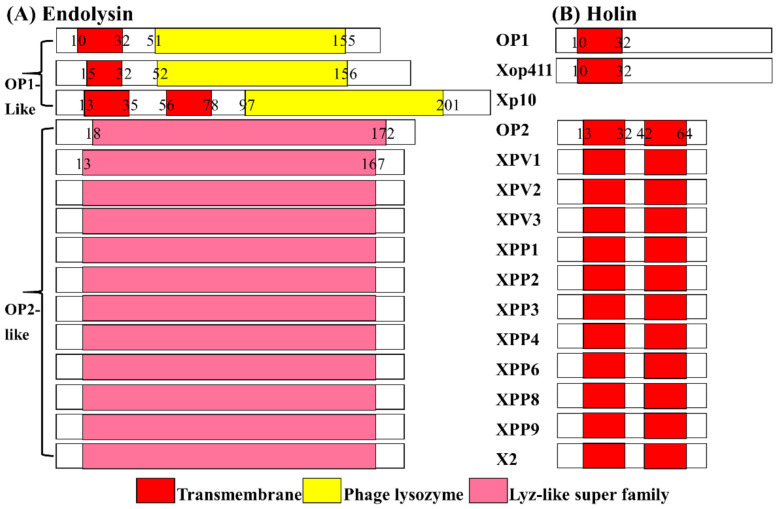
Comparison of lysis system between OP1-like and OP2-like phages of Xoo. (**A**) Endolysin. (**B**) Holin.

Results from this study indicate that phage X2 lysis system is different compared to that of OP1-like phages, but is similar to the one found in OP2-like phages. In general, there are significant differences in the lysis systems when comparing the sequences of endolysins and holins of OP1-like with OP2-like phages. The sequences of endolysins and holins are highly conserved among the OP2- and OP1-like phages but different to each other, while Xp10 is different to both groups (Figure 4). Bioinformatic analyses indicated that endolysin of OP2-like phages do not contain transmembrane domains nor signal peptides. In contrast, endolysins of OP1-like phages Xop411 and OP1 contain at least one N-terminal transmembrane domain, while the endolysin of OP1-like phage Xp10 (with no holin genes) contains two transmembrane domains at the N-terminus, potentially allowing “self-transport” and translocation across the membrane [42,43]. The presence of a single transmembrane domain might potentially allow slow translocation of the endolysin into the periplasm; however, this process might be not rapid enough and thus requires holin-pores for efficient host cell lysis [44]. This may, at least partially, explain the fact that Xop411 and OP1 are predicted to have holins.

Similar to our bioinformatic analyses of the lysis system of Xoo phages, it is well known that significant differences in both morphology and genome sequence between OP1-like and OP2-like phages exist. Wakimoto [45] divided Xoo phages into two groups: OP1 and OP2. OP1-like phages have longer and thinner tails, while OP2-like phages have shorter and thicker tails, with a clear difference in host range as they infect different Xoo strains [23,36]. Similarly, the OP2-like phages are distinctly separated from the OP1-like phages based on their phylogenetics of either whole genomic sequences or their *Terl* gene sequences (Figure 2). Further analysis revealed that the genome of OP1-like phages is linear, while the genome of OP2-like phages is circularly permuted and terminally redundant [6,46]. Although no detailed information is available about the function of endolysin and holin of Xoo phages, the results of this study reveal the diversity and complexity of lysis mechanisms of Xoo phages.

### 3.4. Subcellular Localization and Antibacterial Activity of X2-Lys and X2-Hol

**Figure 5:**
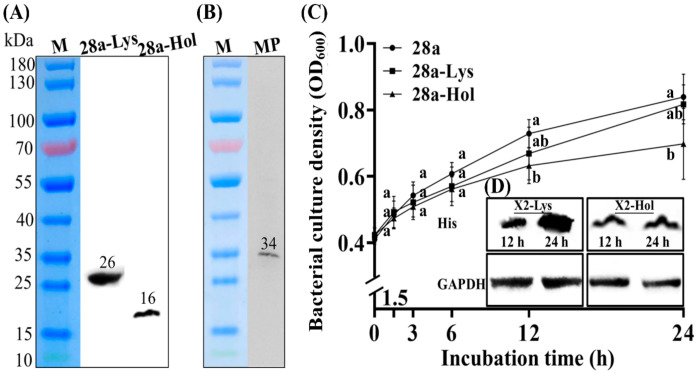
Subcellular localization, expression and antibacterial activity of X2-Lys and X2-Hol. (**A**) X2-Lys and X2-Hol can be expressed in vitro. (**B**) Detection of membrane proteins from *E. coli* BL21 transformed with pET28a-Hol. M: maker; 28a-Lys/Hol: proteins from *E. coli* BL21 transformed with pET28a-Lys/Hol. MP: membrane protein extract. (**C**) Bacterial growth (*n* = 9). (**D**) Western blotting detection of X2-Lys and X2-Hol at 12 h and 24 h post-induction. Anti-His antibody was used to detect for target proteins, i.e: X2-Lys and X2-Hol have His tags; Antibodies against GAPDH was used as a control. Different lowercase letters represent significant difference (*p* < 0.05) in bacterial culture density among the three groups of 28a, 28a-Lys and 28a-Hol at the same time point.

X2-Lys and X2-Hol are 26 kDa and 16 kDa, respectively, as indicated by Western blotting, and is similar to the predicted size of 23.6 kDa and 12.9 kDa, respectively (Figure 5A). This may be due to the fact that the high isoelectric point and His tag fusion have an influence on the migration of the two proteins in SDS PAGE gels [47]. Furthermore, a single band with a molecular weight of about 34 kDa was observed by Western blotting when membranes extracted from *E. coli* BL21 expressing X2-Hol were analyzed, confirming that X2-Hol is a membrane protein. Interestingly, the size of the X2-Hol band is about 2-fold larger than that of the Hol monomer, indicating that X2-Hol formed homodimers in the membrane, which has been reported in a previous study [48].

In order to elucidate the lysis mechanism of phage X2, we cloned the genes encoding X2-Lys and X2-Hol into expression vectors for recombinant protein expression in *E. coli*. We then examined the effect of the expressed proteins on bacterial growth. We observed protein sizes of 26 kDa and 16 kDa by Western blotting, confirming the successful expression X2-Lys and X2-Hol when expression was induced with IPTG. However, X2-Lys showed a greater expression after 24 h of induction compared to 12 h of induction, while no obvious change between 12 h and 24 h in the case of X2-Hol was observed (Figure 5). Furthermore, the expression of X2-Lys did not cause a significant change in bacterial numbers compared to the control after 1.5, 3, 6, 12 and 24 h of induction. In contrast, bacterial growth was unaffected by X2-Hol expression after 1.5, 3 and 6 h of induction, but was significantly inhibited after 12 and 24 h (Figure 5). X2-Lys has no effect on the growth of bacteria, which may be due to the lack of a concerted function with the holin protein. However, it can be inferred that the inhibition of X2-Hol on bacterial growth appears to be a time-dependent process as no obvious change in protein expression was noted.

### 3.5. Changes in Bacterial Morphology Induced by X2-Lys and X2-Hol Expression

**Figure 6:**
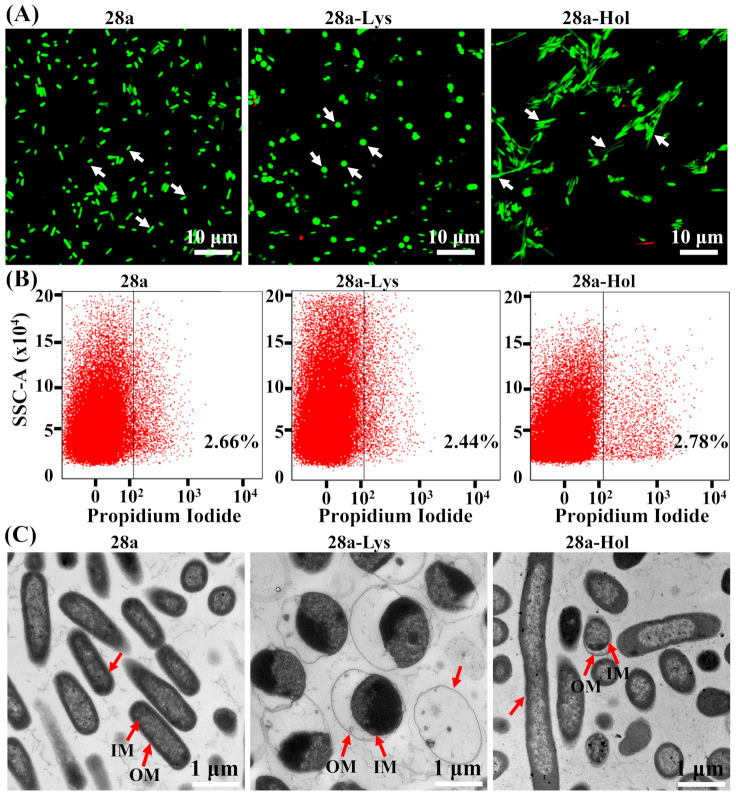
Effect of X2-Lys and X2-Hol on cell morphology at 12 h post-induction. (**A**) Live and dead bacterial staining experiment. (**B**) Flow cytometry scatter plots. Percentage of fluorescent events within gated region is shown in the region on the right. The *x*-axis shows the relative fluorescence intensity and the *y*-axis is the signal from the side scattered light. (**C**) Transmission electron microscopy. Scale bar (white): 1 µm. OM: outer membrane; IM: inner membrane. 28a, empty plasmid control; 28a-Lys: Cells with pET28a-X2-Lys; 28a-Hol: Cells with pET28a-X2-Hol.

Live/dead bacterial staining was carried out using BacLight containing propidium iodide (PI) and SYTO9, usually allowing us to distinguish live cells from dead cells. Intact cells are stained green by the SYTO9 dye but not by PI, which can only penetrate damaged membranes, while the damaged cells exhibit red fluorescence when stained by PI. Fluorescence microscopy showed that only few cells were stained red, implying that most cells were not killed and/or their membranes remained intact when expressing the proteins (Figure 6A). This result is consistent with the data obtained from studying the antibacterial effects of X2-Lys and X2-Hol (Figure 5). In contrast, changes in the *E. coli* cell morphology were observed after 12 h expression of either X2-Lys or X2-Hol. While wild type *E. coli* is rod-shaped, cells expressing X2-Lys became spherical in shape. We observed a third morphology with *E. coli* expressing X2-Hol which exhibited elongated rods (Figure 6A). A similar observation was made by Turnbull et al. [49], who found that endolysin induced a morphological change in rod-shaped *Pseudomonas aeruginosa* cells, which become spherical prior to lysis. Elongated cells are usually due to the inhibition of cell division. A report on *Agrobacterium tumefaciens* cells describes the blocking of cell division by an endolysin [50]; however, in this study, we observe a potential inhibition in cell division by a holin.

Flow cytometry of PI-stained cells allowed the rapid and accurate quantification of the number of live and dead cells, as the dye only penetrates damaged and dead cells, to bind DNA. After 12 h of induction, cell death was similar to control cells (“empty” pET28a plasmid: ~2.7%) (Figure 6B). Here, ~2.4% and ~2.8% of the cells died when expressing X2-Lys and X2-Hol, respectively. Similar to our fluorescence microscopy data, morphological changes from short-rod to spherical or elongated rod were also observed by transmission electron microscopy (TEM). TEM micrographs of *E. coli* with the “empty” pET28a plasmid had an intact cell structure with a high density of biomolecules within the cell. Cells expressing X2-Lys for 12 h showed a separation of the outer membrane structure from the inner membrane, with cells becoming spherical in shape (Figure 6C). In the case of X2-Hol, this effect is less prominent; however, the micrographs show long tubular cells which indicate that holin is negatively impacting cell division.

**Figure 7:**
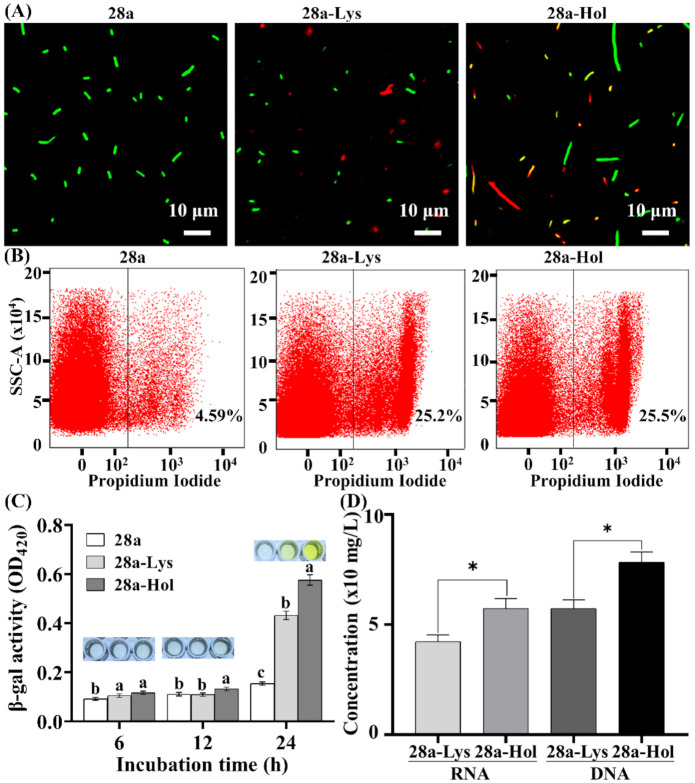
Effect of X2-Lys and X2-Hol on integrity of cell membrane. (**A**) Live and dead bacterial staining after 24 h post-induction. (**B**) Flow cytometry observation after 24 h post-induction. Percentages shown in each image represents the ratio of dead cells to the total cells. (**C**) β-galactosidase activity (OD_420_) after 6, 12 and 24 h post-induction. Different lowercase letters above each bar represent significant difference (*p* < 0.05) in β-galactosidase activity among the three groups of 28a, 28a-Lys and 28a-Hol at the same time point. (**D**) The release of DNA/RNA after 24 h post-induction (*n* = 5). Asterisks (*) above the bar represent significant differences (*p* < 0.05) in RNA or DNA concentrations between 28a-Lys and 28a-Hol groups. 28a-Lys/Hol, 28a carrying X2-Lys/X2-Hol.

Interestingly, we also found that the effect of X2-Lys and X2-Hol on bacterial morphology, integrity of cell membrane and cell death was impacted by the induction time with IPTG. Indeed, similar to the result of the 12 h expression, morphological changes in the *E. coli* cells were also observed after 24 h induction expression of either X2-Lys or X2-Hol. All *E. coli* cells containing the “empty” plasmid pET28a stained green after 24 h of IPTG induction, indicating that they remained viable (Figure 7A). However, a higher percentage of cells stained red after 24 h of IPTG induction compared to that after 12 h of IPTG induction, indicating that a prolonged expression of X2-Lys (with high concentrations according to the Western blotting results) or an extended incubation time with X2-Hol may result in the death of *E. coli* cells (Figure 7A). The death of *E. coli* cells was further confirmed by flow cytometry (Figure 7B). Indeed, the percentage of the damaged cells in cells carrying an “empty” plasmid pET28a was ~4.6%, while the percentage of the damaged cells was ~25.2% and ~25.5% in cells expressing X2-Lys and X2-Hol, respectively, after 24 h of IPTG induction.

In agreement with the results of the antibacterial effect study and cell death count, *E. coli* cells expressing X2-Lys and X2-Hol exhibited a higher degree of membrane damage after 24 h of induction than after 12 h (Figure 7C). Using a colorimetric β-galactosidase release assay, no color change was observed in X2-Lys and X2-Hol compared to the empty plasmid control after 6 and 12 h of IPTG induction. In contrast, after 24 h of protein induction, enzyme release was observed from X2-Lys- and X2-Hol-expressing cells, compared to the control. This result indicated that bacteria were severely damaged after 24 h of induction, with the β-galactosidase activity increasing over time. In addition, X2-Hol seems to result in a stronger release of cytoplasmic content from the cells compared to those expressing X2-Lys. To confirm this observation, we also quantified the amount of DNA and RNA released, which increased significantly compared to the control after 24 h of induction (Figure 7D). Expression of X2-Lys caused an increased release of DNA (57.24 mg/mL) and RNA (42.32 mg/mL) after 24 h of IPTG induction, while expression of X2-Hol resulted in an even higher release of nucleic acids from the cell (DNA: 78.34 mg/mL; RNA: 57.38 mg/mL). The increased release of DNA and RNA caused by X2-Hol compared to X2-Lys is consistent with the results of the antibacterial activity study.

### 3.6. Effects of Co-Expression of X2-Hol and X2-Lys on Cells

**Figure 8:**
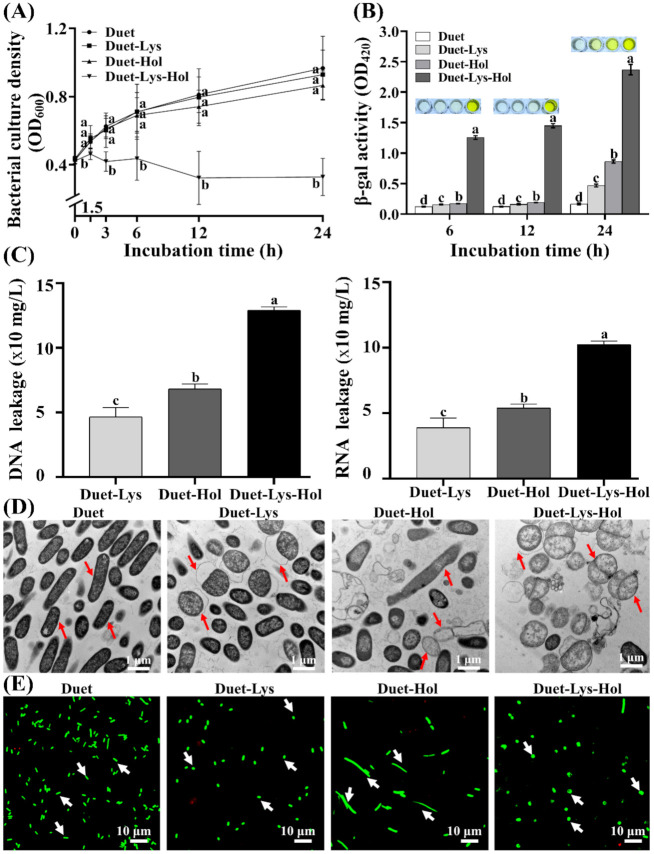
Co-expression of X2-Lys and X2-Hol and its effect on bacterial growth and cell membrane integrity. (**A**) Bacterial growth (*n* = 9). Different lowercase letters represent significant difference (*p* < 0.05) in bacterial culture density among the four groups of Duet, Duet-Lys, Duet-Hol and Duet-Lys-Hol at the same time point. (**B**) β-galactosidase activity (OD_420_) after 6, 12 and 24 h of induction. Different lowercase letters above each bar represent significant difference (*p* < 0.05) in β-galactosidase activity among the four groups of Duet, Duet-Lys, Duet-Hol and Duet-Lys-Hol at the same time point. (**C**) The release of DNA/RNA after 24 h of induction (*n* = 5). Different lowercase letters above each bar represent significant difference (*p* < 0.05) in RNA or DNA leakage among the three groups of Duet-Lys, Duet-Hol and Duet-Lys-Hol. (**D**) Transmission electron microscopic observation after 12 h of induction. (**E**) Fluorescence microscopic observation after 12 h of induction. Duet, empty plasmid; Duet-Lys, Duet carrying X2-Lys; Duet-Hol, Duet carrying X2-Hol; Duet-Lys-Hol, Duet carrying X2-Lys and X2-Hol.

In order to further elucidate the coordinated actions of both endolysin and holin in lysing bacteria, we cloned X2-Lys and X2-Hol into the co-expression plasmid pETDuet-1. This allowed us to examine the effects of expression of both proteins on bacterial growth at different induction times. No significant difference in bacterial growth was observed when expressing X2-Lys and X2-Hol alone. However, bacterial growth was severely inhibited when expressing both proteins together. The co-expression of X2-Lys and X2-Hol caused a ~19%, ~41%, ~55%, ~74% and ~70% reduction in bacterial growth after 1.5, 3, 6, 12 and 24 h of induction, respectively, compared to the control (Figure 8A). This indicates that both X2-Lys and X2-Hol are functional and that they play a synergistic role in bacterial lysis.

The effects of co-expressing X2-Lys and X2-Hol on cell membranes was further measured using β-galactosidase release assays, as described above. Similar to the result from pET28a, no change in color was observed using the empty pETDuet-1 plasmid, indicating cell membranes remained intact and that the β-galactosidase synthesized in the cell is unable to cross the cell membrane [51,52]. Color change (indicating release of the enzyme) was observed in bacteria expressing either X2-Lys or X2-Hol alone after 24 h of induction while bacteria expressing both X2-Lys and X2-Hol showed a much stronger intensity. In particular, the color from cells after 6 h of co-expression is stronger than that of cells expressing the individual proteins, even after 24 h induction. This clearly indicates that co-expression leads to a destabilization of the membrane and release of β-galactosidase (Figure 8B), which explains the strong antibacterial effect that X2-Lys and X2-Hol have together.

It should also be noted that the expression of X2-Lys and X2-Hol alone or together had an impact on the bacterial membrane, as we detected a leakage of cellular components such as nucleic acids (DNA and RNA) from the cells expressing the proteins but not from the controls. We observed a significant difference in the release of nucleic acids of co-expressing strains compared to strains expressing X2-Lys and X2-Hol alone (Figure 8C). Co-expression of X2-Lys and X2-Hol resulted in the increased release of nucleic acids compared to the expression of each protein independently.

The change in morphology and the release of cellular components was further analyzed by fluorescence and transmission electron microscopy of samples obtained after 12 h of induction. Here, *E. coli* with an “empty” pETDuet-1 plasmid displayed the typical rod shape, whereas *E. coli* expressing either endolysin or holin in pETDuet-1 showed similar cell morphology as was observed when carrying out the experiments with pET28a. When both proteins were co-expressed, X2-Lys and X2-Hol caused morphological changes with the cells becoming spherical in shape (Figure 8D,E). Our results are consistent with the findings from Dewey et al. [53], who showed that the expression of endolysin and holin resulted in spherical shaped bacteria. Most cells were observed to be alive according to the life-dead stain. This seemingly contradictory observation may be due to the bactericidal effect caused by the co-expression of endolysin and holin together, leaving only live cells and fragmented small cell debris which cannot retain any dye.

### 3.7. Purified X2-Lys Shows Antibacterial Activity

**Figure 9:**
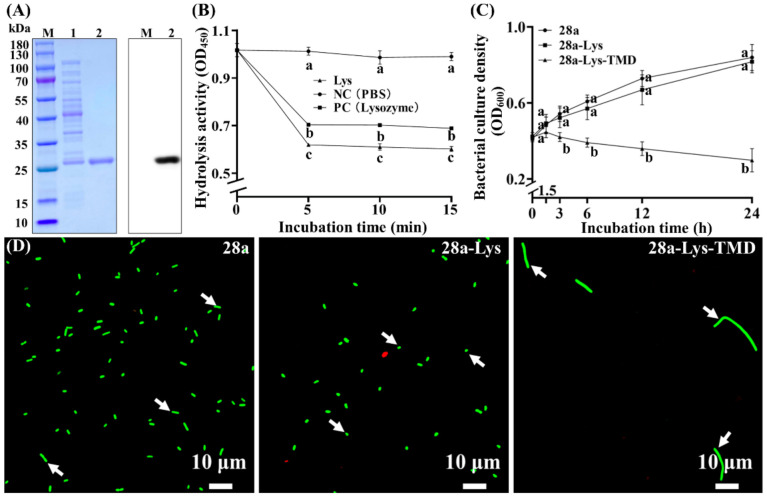
Antibacterial activity of X2-Lys with the help of TMD. (**A**) SDS-PAGE and Western Blotting of the purified X2-Lys. Lane M: maker; Lane 1: unpurified X2-Lys; Lane2: purified X2-Lys. (**B**) Hydrolytic activity of peptidoglycan by purified X2-Lys. NC: negative control; PC: positive control. Mean ± SD are shown (*n* = 3). Different lowercase letters represent significant difference (*p* < 0.05) in hydrolytic activity among the three groups of X2-Lys, PBS (NC) and lysozyme (PC) at the same time point. (**C**) Bacterial growth after 0, 1.5, 3, 6, 12 and 24 h of IPTG induction (*n* = 9). Different lowercase letters represent significant difference (*p* < 0.05) in bacterial culture density among the three groups of 28a, 28a-Lys and 28a-Lys-TMD at the same time point. (**D**) Fluorescence microscopic observation after induction of 12 h. 28a, empty control plasmid; 28a-Lys, 28a plasmid carrying X2-Lys; 28a-Lys-TMD, 28a plasmid carrying X2-Lys and a transmembrane domain. Bacteria with different morphology were marked with white arrows.

As endolysins have been reported to possess antibacterial properties, we also tested if X2-Lys exhibited a similar activity. We first purified the protein by using a Ni-NTA resin and obtained a single band on the SDS PAGE gel, which produced a strong signal using anti-hexa-His antibodies to detect the fusion tag (Figure 9A).

In agreement with the results of previously published studies [54,55], our data shows that X2-Lys, on its own, has no effect on the Gram-negative strain Xoo. This aligns well with the results obtained from our bioinformatic analysis which indicated that endolysin contains no transmembrane domain nor signal peptide. Hence, X2-Lys itself may not be able to penetrate the bacterial outer membrane to hydrolyze the bacterial cell wall. Previous reports have shown that endolysin added from the outside results in cell death, which may be due to two reasons. One is that endolysin can directly attack the molecular structure of Gram-positive bacteria, which have no outer membrane [56,57]. The other is that endolysins with antibacterial effects in vitro are generally applied to bacteria together with outer membrane permeabilizers [58,59,60], as Gram-negative bacteria have an outer membrane, preventing antibacterial effects [54]. In order to assist X2-Lys to reach its target, the peptidoglycan layer, EDTA was used in this study to chelate divalent ions that stabilize the outer membrane via electrostatic interactions, mainly occurring among LPS molecules [61]. While the negative control (PBS with EDTA) shows no change in bacterial numbers regardless of the incubation time, the addition of the X2-Lys protein under these conditions resulted in a strong antimicrobial effect, causing an approximate 40% reduction in bacterial numbers after 5, 10 or 15 min. The positive control (lysozyme) caused a ~30% reduction in bacterial numbers over the same period (Figure 9B), demonstrating the effectiveness of endolysin X2-Lys, given that the target can be reached.

### 3.8. Potential Multiple Roles of the Transmembrane Domain in X2-Lys for Transport and Division Inhibition

Compared to the pET28a empty plasmid in the control, bacterial numbers were unaffected by X2-Lys expression in *E. coli* at all tested induction times and the expression of X2-Lys with a transmembrane domain (28a-Lys-TMD) after 1.5 h of incubation. However, bacterial numbers were significantly reduced when pET28a-Lys-TMD was expressed for 3, 6, 12 and 24 h, which caused a 22.3%, 35.7%, 50.8% and 64.5% reduction in bacterial numbers, respectively (Figure 9C). Fluorescence microscopy showed that the expression of X2-Lys alone caused a morphological change from short-rod to spherical, while expression of the X2-Lys with a transmembrane domain in *E. coli* caused the bacteria to appear as long rods. This curious effect may be the result of Lys-TMD inhibiting the division of bacterial cells (Figure 9D). Interestingly, Attai et al. (2017) also found that the transmembrane domain of phage endolysin plays a key role in cell elongation of *Agrobacterium tumefaciens*, leading to the largest possible phage release of bacteria, benefiting the phage [50]. Therefore, we propose that the transmembrane domain plays a similar role in the case of X2-Lys.

Previous experiments show that X2-Lys has the ability to lyse bacteria with the help of holin, which contains two transmembrane domains. Hence, we raised the question of whether the addition of a transmembrane domain to X2-Lys would mediate the translocation of the protein through the cell membrane in order to induce lysis. We observed that the number of bacteria expressing an endolysin with a TMD resulted in a massive decrease compared to the control. We interpret this as the efficient release of Lys into the periplasmic space mediated by the presence of a TMD. Although holin and the protein with a TMD may have a similar role with regard to transmembrane transport of Lys, the observed differences in morphology when co-expressed. This indicates different mechanisms of the two pathways. We believe that the role of the TMD in cell lysis is mainly to facilitate Lys transport but also to inhibit bacterial cell division.

## 4. Conclusions

**Figure 10:**
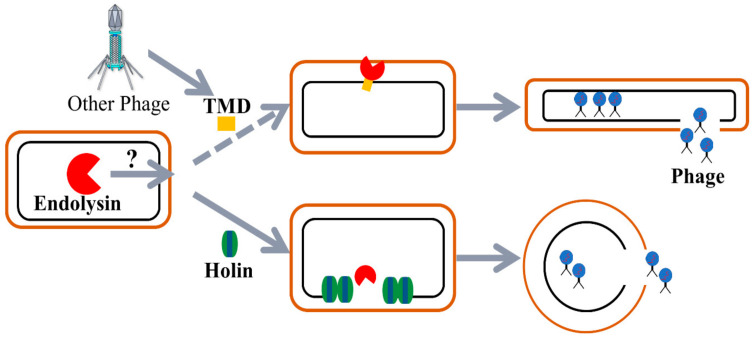
Transport of the endolysin mediated by holin or the TMD, which can result in different morphological changes of the host cells.

This study identified and systematically analyzed the holin-endolysin lysis system of the OP2-like phage X2, which has the ability to infect and inactivate the most important bacterial rice pathogen. In silico analysis revealed that the endolysin of OP2-like phages lack a transmembrane domain (TMD), suggesting X2-Lys alone is unable to achieve bacterial lysis and requires the type II holin X2-Hol, which exhibits substantial differences compared to the holin found in Xoo OP1-like phages. The requirement for holin in the lysis process together with X2-Lys was demonstrated by the negative impact that co-expression had on bacterial cell numbers, which also resulted in membrane damage and the leakage of intracellular content, as compared to X2-Lys and X2-Hol alone. Interestingly, we found that the TMD facilitated the transport of the endolysin via the Sec pathway in phage AP1 of the rice pathogen *A. oryzae*. Morphological changes were observed in *E. coli* cells, when co-expressing Lys with holin or with a TMD. Our study revealed that X2-Lys of Xoo phage can exert its function when a TMD is present and plays a dual role: One is the transmembrane transport while the other is the inhibition of cell division (Figure 10).

## Figures and Tables

**Table 1:** Plasmids used in this study.

Plasmids	Description	Sources
pET28a	Km^R^; cloning vector	Novagen
pET28a-Lys	Km^R^; recombinant expression vector with a X2-Lys	This study
pET28a-Hol	Km^R^; recombinant expression vector with a X2-Hol	This study
pET28a-Lys-TMD	Km^R^; recombinant expression vector with a X2-Lys-TMD	This study
pETDuet-1	Amp^R^; cloning vector	Laboratory collection
pETDuet-Lys	Amp^R^; recombinant expression vector with a X2-Lys	This study
pETDuet-Hol	Amp^R^; recombinant expression vector with a X2-Hol	This study
pETDuet-Lys-Hol	Amp^R^; recombinant expression vector with a X2-Lys and X2-Hol	This study

Amp^R^/Km^R^: ampicillin-/kanamycin-resistant. TMD: transmembrane domain.

**Table 2:** Primers used in this study.

Primer Name	Nucleotide Sequence (5′–3′)	Characterization
28a-Lys-F	CGGGATCCATGTCAACTGAGACTGTCGCAG (B)	Gene of Lys from X2 phage
28a-Lys-R	CCCAAGCTTTACGCTCAGTCCAAACAGGCG (H)	
28a-Hol-F	CGGGATCCATGACCCCTGAACCTCGTAATATCG (B)	Gene of Hol from X2 phage
28a-Hol-R	CCCAAGCTTGCCTGCATTGTTTGCGCC (H)	
TMD-F	CCCAAGCTTAGCCTCGGCAACTGGC (H)	Gene of TMD
TMD-R	CCGCTCGAGTGACCACCCCTCTCGCC (X)	
Duet-Lys-F	AACTGCAGATGTCAACTGAGACTGTCGCAG (P)	Gene of Lys from X2 phage
Duet-Lys-R	CCCAAGCTTTACGCTCAGTCCAAACAGGCG (H)	
Duet-Hol-F	CTATACATATGATGACCCCTGAACCTCGTAATATCG (N)	Gene of Hol from X2 phage
Duet-Hol-R	GAAGATCTTCAGCCTGCATTGTTTGCGCC (Bg)	

Underlined nucleotides indicate restriction enzyme recognition sites in parentheses (B: BamHI; H: HindIII; X: XhoI; P: PstI; N: NdeI; Bg: BglII).

**Table 3:** Comparison of the available genomes of *Xanthomonas oryzae* pv. *oryzae* phages from the GenBank database.

Type	Name	Length (bp)	G+C Content (%)	Number of ORF	Accession No.
OP1-					
	OP1	43,785	51	59	AP008979.1
	Xop411	44,520	52	60	NC_009543.1
	Xp10	44,373	52	60	AY299121.1
OP2-					
	OP2	46,643	61	62	AP008986.1
	XPP1	46,195	62	73	MG944227
	XPP2	46,204	61	72	MG944228
	XPP3	46,201	62	78	MG944229
	XPP4	46,200	62	73	MG944230
	XPP6	46,204	61	72	MG944231
	XPP8	46,184	62	74	MG944232
	XPP9	46,201	61	80	MG944233
	XPV1	46,503	60	77	MG944234
	XPV2	45,969	64	74	MG944235
	XPV3	47,046	60	76	MG944236
	X2	45,966	61	74	MW435566

Data obtained from GenBank.

## Data Availability

The data presented in this study are available within the article.

## Supplementary Materials

The following are available online at https://www.mdpi.com/article/10.3390/v13101949/s1, Table S1: Phage X2 genome annotation analysis GenBank.
