# Supplementary material for: The Holin-Endolysin Lysis System of the OP2-Like Phage X2 Infecting Xanthomonas oryzae pv. oryzae
Source: Viruses. 2021 Sep 28;13(10):1949. doi: 10.3390/v13101949 (PMC8541568; doi:10.3390/v13101949)
Supplement: Supplementary file 1 [file viruses-13-01949-s001.zip › viruses-1374043-supplementary.pdf]

Table S1 Phage X2 genome annotation analysis.

ORF	Coding region (bp)	Strand	Protein size (aa)	Putative function	Best match[a]	Accession no.
1	2-661	+	219	Transposon or IS	XPP1 (98%)	QRI46302
2	670-2085	+	471	hypothetical protein	OP2 (99%)	QRI46303
3	2645-2127	-	172	hypothetical protein	OP2 (100%)	QRI46304
4	2862-2635	-	75	Phage protein	OP2 (100%)	QRI46305
5	3383-2859	-	174	Membrane-bound lytic murein transglycosylase D precursor	XPV2 (98%)	QRI46306
6	3753-3373	-	126	hypothetical protein	OP2 (99%)	QRI46307
7	3988-3746	-	80	hypothetical protein	XPV2 (95%)	QRI46308
8	4598-3981	-	205	hypothetical protein	XPV2 (85%)	QRI46309
9	4777-4595	-	60	hypothetical protein	XPV1 (97%)	QRI46310
10	5168-4788	-	126	Phage Holliday junction resolvase RusA	XPV2 (97%)	QRI46311
11	5350-5481	+	43	hypothetical protein	/	QRI46312
.12	6122-5535	-	195	hypothetical protein	XPP1 (97%)	QRI46313
13	6288-6139	-	49	hypothetical protein	XPP1 (100%)	QRI46314
14	6974-6342	-	210	hypothetical protein	XPP1 (99%)	QRI46315
15	7465-7502	-	137	hypothetical protein	XPP1 (97%)	QRI46316
16	7616-7452	-	54	hypothetical protein	XPP9 (94%)	QRI46317
17	8815-7613	-	400	hypothetical protein	OP2 (95%)	QRI46318
18	9373-9840	+	155	Phage integrase	XPP9 (98%)	QRI46319
19	9837-11291	+	484	Phage terminase, large subunit	XPP1 (99%)	QRI46320
20	11334-12932	+	532	hypothetical protein	XPV2 (99%)	QRI46321
21	12973-13770	+	265	Phage minor capsid protein	XPV1 (97%)	QRI46322
22	13926-15023	+	365	Phage head and packaging protein	XPP3 (92%)	QRI46323
23	15037-15570	+	177	putative structural protein	OP2 (100%)	QRI46324
24	15604-16623	+	339	putative structural protein	OP2 (96.76%)	QRI46325
25	16690-17175	+	161	hypothetical protein	OP2 (90.00%)	QRI46326
26	17188-17691	+	167	hypothetical protein	OP2 (99.40%)	QRI46327
27	17688-18194	+	168	putative RNA polymerase	OP2 (98.21%)	QRI46328
28	18191-18616	+	141	hypothetical protein	XPP1 (100.00%)	QRI46329
29	18613-19140	+	175	hypothetical protein	XPP1 (98.29%)	QRI46330
30	19164-20648	+	494	hypothetical protein	XPV1 (98.58%)	QRI46331

31	20661-21095	+	144	hypothetical protein	OP2 (97.22%)	QRI46332
32	21346-21146	-	66	hypothetical protein	XPV1 (53.70%)	QRI46333
33	21873-21343	-	176	hypothetical protein	XPV1 (85.70%)	QRI46334
34	22417-21860	-	185	hypothetical protein	XPV1 (96.76%)	QRI46335
35	22593-22417	-	58	hypothetical protein	XPP1 (96.55%)	QRI46336
36	22824-23735	+	303	hypothetical protein	/	QRI46337
37	24448-23933	-	171	hypothetical protein	OP2 (98.83%)	QRI46338
38	24636-24445	-	63	hypothetical protein	XPV1 (95.24%)	QRI46339
39	24800-24633	-	55	hypothetical protein	XPP1(100.00%)	QRI46340
40	25026-24823	-	67	hypothetical protein	OP2 (98.51%)	QRI46341
41	25276-25037	-	79	hypothetical protein	XPP1 (98.73%)	QRI46342
42	27357-25381	-	658	putative DNA polymerase I	OP2 (98.63%)	QBA09445.<br>1
43	27801-27421	-	126	hypothetical protein	XPP1 (98.41%)	QRI46343
44	29713-27869	-	614	putative phage helicase	OP2 (98.86%)	QRI46344
45	29998-29807	-	63	hypothetical protein	XPP1(100.00%)	QRI46345
46	30186-29995	-	63	hypothetical protein	XPP1 (98.41%)	QRI46346
47	30371-30183	-	62	hypothetical protein	XPV1(100.00%)	QRI46347
48	30571-30368	-	67	hypothetical protein	XPV1 (88.06%)	QRI46348
49	30812-30561	-	83	hypothetical protein	XPV2 (89.16%)	QRI46349
50	31228-30812	-	138	hypothetical protein	XPP8 (92.03%)	QRI46350
51	31699-31457	-	80	hypothetical protein	XPV3 (95.00%)	QRI46351
52	31839-31696	-	47	hypothetical protein	XPP1 (95.74%)	QRI46352
53	32137-31952	-	61	hypothetical protein	XPV2 (98.36%)	QRI46353
54	32382-32134	-	82	hypothetical protein	OP2 (98.78%)	QRI46354
55	32930-32379	-	183	hypothetical protein	XPP1 (98.91%)	QRI46355
56	33387-32935	-	150	hypothetical protein	OP2 (100.00%)	QRI46356
57	33808-33419	-	129	hypothetical protein	XPP1 (100.00%)	QRI46357
58	34097-33825	-	90	hypothetical protein	XPV1 (97.78%)	QRI46358
59	34421-34107	-	104	hypothetical protein	XPP1 (98.08%)	QRI46359
60	34655-34446	-	69	hypothetical protein	XPP4 (100.00%)	QRI46360
61	37236-34723	-	837	hypothetical protein	XPV2 (99.52%)	QRI46361
62	37382-37248	-	44	hypothetical protein	/	QRI46362

63	37409-37525	+	38	hypothetical protein	XPP3 (73.68%)	QRI46363
64	37750-38133	+	127	hypothetical protein	OP2 (100.00%)	QRI46364
65	38130-38531	+	133	hypothetical protein	XPP9 (98.50%)	QRI46365
66	38605-39219	+	204	putative tail fiber protein	OP2 (99.02%)	QRI46366
67	39389-39928	+	179	hypothetical protein	XPP1 (99.44%)	QRI46367
68	39952-40347	+	131	hypothetical protein	OP2 (100.00%)	QRI46368
69	40351-41025	+	224	hypothetical protein	OP2 (100.00%)	QRI46369
70	41029-42864	+	611	Phage tail protein	OP2 (98.04%)	QRI46370
71	42869-43768	+	299	hypothetical protein	XPP1 (99.33%)	QRI46371
72	43752-43994	+	80	hypothetical protein	OP2 (100.00%)	QRI46372
73	43987-44649	+	220	putative baseplate protein	OP2 (96.82%)	QRI46373
74	44667-45941	+	424	hypothetical protein	XPP1 (100.00%)	QRI46374

"/" means no significant matched in GenBank. [a]all matched phages belong to the *Xanthomas* phage.
